# Artificial intelligence and the diagnosis of oral cavity cancer and oral potentially malignant disorders from clinical photographs: a narrative review

**DOI:** 10.3389/froh.2025.1569567

**Published:** 2025-03-10

**Authors:** Payam Mirfendereski, Grace Y. Li, Alexander T. Pearson, Alexander Ross Kerr

**Affiliations:** ^1^ Departmment of Oral and Maxillofacial Pathology, Radiology, and Medicine, New York University College of Dentistry, New York, NY, United States; ^2^ Department of Medicine, Section of Hematology/Oncology, University of Chicago Medical Center, Chicago, IL, United States

**Keywords:** oral cavity cancer, oral squamous cell carcinoma, oral potentially malignant disorders, artificial intelligence, deep learning, computer vision, image analysis, clinical photographs

## Abstract

Oral cavity cancer is associated with high morbidity and mortality, particularly with advanced stage diagnosis. Oral cavity cancer, typically squamous cell carcinoma (OSCC), is often preceded by oral potentially malignant disorders (OPMDs), which comprise eleven disorders with variable risks for malignant transformation. While OPMDs are clinical diagnoses, conventional oral exam followed by biopsy and histopathological analysis is the gold standard for diagnosis of OSCC. There is vast heterogeneity in the clinical presentation of OPMDs, with possible visual similarities to early-stage OSCC or even to various benign oral mucosal abnormalities. The diagnostic challenge of OSCC/OPMDs is compounded in the non-specialist or primary care setting. There has been significant research interest in technology to assist in the diagnosis of OSCC/OPMDs. Artificial intelligence (AI), which enables machine performance of human tasks, has already shown promise in several domains of medical diagnostics. Computer vision, the field of AI dedicated to the analysis of visual data, has over the past decade been applied to clinical photographs for the diagnosis of OSCC/OPMDs. Various methodological concerns and limitations may be encountered in the literature on OSCC/OPMD image analysis. This narrative review delineates the current landscape of AI clinical photograph analysis in the diagnosis of OSCC/OPMDs and navigates the limitations, methodological issues, and clinical workflow implications of this field, providing context for future research considerations.

## 1 Introduction

With a GLOBOCAN-estimated incidence of 389,485 cases and 188,230 mortalities in 2022, oral cavity and lip cancer remains a significant global source of cancer burden (1). Greater than 90% of oral cavity cancers are squamous cell carcinomas (OSCC), and in contrast to the relatively indolent natural history of cutaneous SCC, OSCCs are typically aggressive. The 5-year survival rate of OSCC drops markedly with advanced stage diagnosis, underscoring the need for continued research on both technological and institutional mechanisms of facilitating early diagnosis. Clinical suspicion of OSCC necessitates prompt tissue biopsy and histopathological examination to definitively confirm the diagnosis. Advanced stage OSCC often has an ominous clinical presentation, a clinical diagnosis with high correlation to the definitive diagnosis. Early stage OSCC, however, is less overt in its clinical presentation, with clinical features that overlap with those of numerous oral mucosal disorders. As such, the “risk stratification” or triage of patients presenting with abnormal mucosal findings can be challenging, particularly in a non-expert primary care setting.

The natural history of oral carcinogenesis varies from patient to patient, and a majority of patients who develop OSCC have a history of “premalignant” disease, variably manifesting with oral mucosal abnormalities that carry an increased risk of OSCC development known as oral potentially malignant disorders (OPMDs) (2). OPMDs are “clinical” diagnoses rendered chairside by the clinician and based upon history and clinical examination findings that correlate to eleven different disorders (leukoplakia, erythroplakia, proliferative verrucous leukoplakia, oral submucous fibrosis, oral lichen planus, oral lichenoid lesions, oral graft-vs. host disease, oral lupus erythematosus, actinic keratosis, palatal lesions in reverse smokers, and dyskeratosis congenita). Oral leukoplakia (OL), a commonly encountered OPMD, is a diagnosis of exclusion made by ruling out benign diseases with overlapping features. There are two subcategories of OL, homogeneous and non-homogeneous leukoplakia. Homogeneous leukoplakia is the more prevalent form and typically presents as a homogeneous flat white plaque with well-delineated margins. Non-homogeneous leukoplakia is less prevalent and can present as a white plaque with variable surface topography (i.e., nodular or verrucous), or a mixed or even speckled red and white plaque (i.e., erythroleukoplakia). Non-homogeneous leukoplakia has a higher propensity to become cancer. Similarly, the other OPMDs have unique clinical features and categorization.

Taken collectively, the sheer number of permutations in clinical presentation across the spectrum of OPMDs (and benign diseases with overlapping features) augment the complexity of decision-making both in a primary and in an expert clinical setting. In a primary care setting, where OSCC and OPMDs have a low prevalence (approximately 2%), clinicians, such as general dentists, typically lack experience in the triage of patients presenting with abnormal mucosal findings. Primary care clinicians often use the term “suspicious”, a crude triage term that typically triggers the important decision to refer a patient for further diagnostic evaluation by an expert. General dentists have been found to lack an analytical and consistent decision-making strategy in the differentiation of malignant, premalignant, and benign oral epithelial lesions based on clinical cues (3). This can lead to both over- and under-diagnosis and inefficient referral of patients. Various adjunctive tools, including light-based detection systems and brush cytology, have been systematically studied over the past few decades for their capacity to improve triage accuracy of patients with mucosal abnormalities “suspicious” for OPMDs/OSCC. Yet they too can lead to both over- and under-diagnosis of patients (4, 5). Furthermore, some of these adjunctive tools are accompanied by challenges such as costliness and limited availability that have precluded their widespread incorporation into clinical practice.

In a secondary care setting, such as an oral medicine service, patients referred with “suspicious” mucosal findings are re-examined. Expert corroboration of the clinical diagnosis of OSCC/OPMD will typically trigger tissue biopsy to establish a definitive “gold standard” histopathological diagnosis that is the current basis for management of this patient population (6). Patients with definitive diagnosis of OSCC are typically referred to tertiary oncology care centers for treatment. Patients with OPMDs may receive variable histopathological diagnoses ranging from OSCC to oral epithelial dysplasia (OED, with various grades or severity) to non-dysplastic benign diagnoses. The risk for cancer development of patients with non-malignant diagnoses is highly variable and a number of predictive factors for cancer development in patients with OL have been identified and reviewed elsewhere (some examples include the clinical phenotype of non-homogenous OL > homogenous OL, the histological diagnosis of high > low grade OED, and the presence of biomarkers such as aneuploidy of loss of heterozygosity (7), and the composite of such predictors can carry different implications for both cancer risk assessment and management decisions. Irrespective of management decisions, all patients with a history of OSCC and OPMDs require close monitoring.

In recent years, there has been a surge in research on using artificial intelligence (AI) in the diagnosis and prognostication of a massive range of medical and dental conditions, including OSCC/OPMDs. AI encompasses myriad functionalities that reproduce tasks previously attributed only to human cognition. Computer vision, a field of AI dedicated to the interpretation and understanding of visual data, has been increasingly applied to medical diagnostics. Computer vision tasks such as object detection, image segmentation, and image classification seek to replicate or improve upon human image analysis. Various research groups have duly examined the effectiveness of AI systems in the diagnosis of OSCC/OPMDs based on clinical photography as well as other imaging modalities.

Given the complexity of triaging patients with abnormal oral mucosal disease, the application of AI to clinical photographs has been proposed as an easily accessible and efficient diagnostic adjunct for OSCC/OPMDs. The feasibility of such real-time chairside triage from clinical photographs could engender a consequential paradigm shift to both an earlier diagnosis of patients with OSCC and the identification of patients with OPMDs, particularly those at higher risk for cancer development. This, in turn, could lead to a more efficient flow of patients from primary care into expert care, thus reducing overall disease burden (i.e., morbidity and mortality). Along with its expanding capacities and promise in this domain, however, computer vision presents its own unique challenges related to methodology and clinical applicability.

The studies that have applied AI clinical photograph analysis to OSCC/OPMDs diagnosis have had varying goals, ranging from a binary classification of patients with abnormal mucosal findings to facilitate referral of patients from a primary to expert care setting, to more complex multi-class discriminative tasks (i.e., the ability to develop *a priori*tized differential diagnosis or to assign cancer risk assessment categories). Several systematic reviews have thus far delineated the scope of AI in the diagnosis of OSCC and/or OPMDs based on clinical photographs. This literature has space to benefit from a more comprehensive scrutiny of the methodological and workflow implications and the limitations of AI clinical photograph analysis. The purpose of this narrative review is to appraise the status of AI clinical photograph analysis in the diagnosis of OSCC/OPMDs and elucidate its current methodological implications and clinical applicability concerns and, in so doing, to steer future research toward more streamlined and standardized applications of computer vision in the chairside diagnosis of OSCC/OPMDs. This review seeks the collaborative perspective of both data engineers well-versed in the technical aspects of AI algorithms and expert clinicians, such as Oral Medicine specialists, engaged in the diagnosis and management of patients with oral mucosal diseases. It will consist of an overview of the current terminology and standards in AI image analysis, a description of salient methodological findings from the extant studies on OSCC/OPMDs photograph analysis, and a discussion about these studies’ methodological and clinical applicability implications.

## 2 Section 1: overview of AI and image analysis

Artificial intelligence (AI), which involves the generation of human-like learning and tasks by machines, encompasses an ever-broadening set of disciplines and branches. *Computer vision* is the discipline of AI dedicated to the analysis and interpretation of visual data such as images and videos and forms the foundation of modern image analysis (8). This field has greatly benefited from developments in both machine learning and deep learning techniques.

*Machine learning*, a core discipline of AI, uses computational algorithms to create models that make predictions and “learn” from patterns within training data while improving their performance without explicit programming (9). In the context of image analysis, machine learning methods such as decision trees, support vector machines, k-nearest neighbors, and simple neural networks have been applied to tasks such as image classification and object detection. However, conventional machine learning approaches often require human expertise for feature identification, extraction, and weighting in order to build a predictive model; this “feature engineering” process may be labor-intensive and less robust than desired in interpreting complex visual data with large variations (8, 10).

In recent years, *deep learning* has replaced conventional machine learning as the standard in image analysis (10). *Deep learning* is a specialized branch of machine learning that utilizes artificial neural networks with many (“deep”) layers that can learn increasingly complex image features, from edges to shapes to entire objects and scenes (9). Deep convolutional neural networks (CNNs) have the capacity to automatically extract discriminative features from data and adjust their weights through iterative backpropagation, refining the model's predictive performance without requiring manual feature extraction. With each year, even more advanced model architectures continue to emerge. For example, vision transformers treat images as sequences of patches, allowing for global context understanding and often outperforming CNNs on some tasks. Generative adversarial networks can learn to generate highly realistic synthetic images, enabling novel applications in synthetic image data generation and augmentation.

The application of machine learning and deep learning in image analysis can be categorized into supervised, unsupervised, semi-supervised, or self-supervised approaches (9). In *supervised learning*, the model is trained on labeled or annotated data and used to predict already known outcomes. In medical image analysis, a supervised model might be trained on a dataset where each image is labeled with a known diagnosis. These labels are the “ground-truth.” During training, the model learns to associate image features with these labels through the following process: for each given image, the model outputs a predicted label (e.g., a diagnosis). This prediction is compared with that image's “ground-truth” label to gauge the level of error. The model uses this feedback to adjust its internal parameters, gradually improving its predictive performance. The effectiveness of supervised learning relies heavily on the quality and quantity of labeled data. However, generating “ground-truth” labels for an image dataset often requires expert knowledge (e.g., from medical professionals) and can be costly in terms of time, money, and manual labor.

*Unsupervised learning* works with unlabeled data, aiming to recognize patterns, cluster similar images, or identify anomalies. In image analysis, unsupervised learning is often used for dimensionality reduction, exploratory analysis of large datasets, and as a precursor to developing more specialized models. *Semi-supervised learning* bridges supervised and unsupervised approaches by leveraging a subset of labeled data within a larger pool of unlabeled data. This method is particularly useful when labeled data is scarce or expensive to obtain. *Self-supervised learning* leverages large amounts of unlabeled data to learn meaningful representations of the data's structure. This technique has proven effective in developing “foundation models” for medical imaging. For example, pathology image foundation models have learned representations of general pathology visual features that can be extracted and applied to specific datasets (i.e., of a specific cancer). These pre-learned features serve as powerful inputs for training new models on specific diagnostic tasks, often resulting in improved performance using much smaller labeled datasets compared to training from scratch.

In practice, supervised learning remains the most common approach in medical image analysis due to its direct applicability. However, semi-supervised and self-supervised techniques are gaining popularity, especially in scenarios with limited labeled data. Self-supervised learning, in particular, typically requires substantial amounts of unlabeled data to be effective, which can be an advantage in fields where unlabeled data is abundant.

The workflow for AI model development comprises various steps (Table 1). Images used for AI analysis typically undergo several processing procedures prior to their input to AI models. *Image quality control* involves curation of datasets to maintain quality, which can include elimination of images affected by blurriness, noise, or artefacts. *Image normalization* refers to the calibration of images by standardizing parameters such as pixel intensity, color saturation, and size to uniform scales. *Dataset harmonization* encompasses steps applied to organize the dataset and prepare it for algorithmic input, such as removal of duplicate images, elimination of sensitive and irrelevant information, and unique and standardized labeling. *Data augmentation* is a computational solution to limited training data and refers to the generation of variants of original training images based on techniques such as rotation, flipping, cropping, scaling, translation, color transformations, and noise injection. Data augmentation in theory enhances a model's invariance, or its resistance to artefactual variations or perturbation of data, and it may also increase its generalizability to outside data.

**Table 1:** Terminology of workflow steps and model components in AI image analysis.

Term	Definition
Image quality control	Application of inclusion and exclusion criteria to images to maintain image quality, such as elimination of images affected by blurriness, noise, or artefacts
Image normalization	Adjustment of image parameters such as pixel intensity, color saturation, or image size to a uniform scale for comparability
Dataset harmonization	Cleaning and preparing the dataset for the training process by removing duplicate images, eliminating sensitive information, standardizing labeling, and assigning unique identifiers
Data augmentation	Generation of variants of original training images based on techniques such as rotation, flipping, cropping, scaling, translation, color transformations, and noise injection
Object detection	Localization of an object within an image, typically by drawing a bounding box
Semantic segmentation	Demarcation of all pixels associated with a particular class with no distinction between individual objects encountered in that class
Instance segmentation	Demarcation of pixels associated with a particular class with distinction of each individual object encountered in that class
Classification	Assignment of categories to images at the binary level (2 categories) or multi-class level (3 or more categories)
Training	Process of teaching a model to recognize data patterns and make predictions from input data
Transfer learning	Pre-training of a model on a large, pre-established image dataset followed by fine-tuning with the study dataset
Validation	Refinement of model parameters based on performance on training data
Testing	Verification of a trained model's performance on unseen data
Internal validation	Verification of a trained model's performance on unseen data split from the original internal dataset
External validation	Verification of a trained model's performance on unseen data from a different temporal or geographic source
Explainability	Methods seeking to provide insight into the decision-making processes of a model
Uncertainty quantification	Methods seeking to quantify a model's uncertainty level with its decisions

Different algorithms allow the completion of several important image analysis tasks, including object detection, segmentation, and classification (9). *Object detection* is the localization of a specific object in an image via generation of a bounding box around the object (also known as “annotation”). Segmentation is the pixel-level delineation or demarcation of a specific object or objects in an image. *Semantic segmentation* produces a single outline or “mask” for all cases in a particular category (i.e., OL) present in the image, while *instance segmentation* produces separate masks for each case in the category. *Classification* is the assignment of categories to images, and it can be either binary, which discriminates between two classes, or multi-class, which discriminates between three or more classes.

The development of a typical AI image analysis model incorporates the phases of training, validation, and testing (11). During *training*, the model iteratively processes the data, learning to recognize patterns and make predictions by repeatedly adjusting its internal parameters to minimize errors on labeled examples. While models typically require large datasets to learn complex patterns and generalize well, limited data availability is a frequent challenge in the medical realm (9). To address this issue, researchers often employ *transfer learning*, a technique that involves pre-training a model on a larger non-study (often non-medical) dataset, followed by fine-tuning with the smaller, specialized medical study dataset.

In the domain of AI, *validation* refers to a phase in the training process used to refine the model's parameters based on its prediction errors during training iterations, which differs from the common interpretation in the medical field of validation as a verification of performance (11). In AI, *testing* is the term used for the verification of the model's performance on unseen data. However, in medical AI literature, the terms *internal validation* and *external validation* have been used to describe the verification of a model's performance on unseen data derived either internally, i.e., split from the original dataset, or externally, i.e., obtained from a different time or location than the original dataset (11). These terms align more closely with the AI concept of testing, emphasizing the importance of assessing model performance on independent datasets.

The purpose of internal and external validation is to assess the generalizability of the model beyond the training data to the population of interest. Overfitting occurs when a model is overly adapted to the training data and performs poorly on unseen data (12), while underfitting refers to a model that inadequately processes relationships in training data and thus performs poorly with both training and testing data. As with non-AI diagnostic and predictive models, an external validation with an unbiased dataset reflective of the population of interest constitutes the superior means of verifying a model's generalizability and clinical applicability (11).

Evaluation of AI performance is possible through various established metrics, which can differ based on the specific task performed. The main metrics in classification tasks include the following (9):

Accuracy: percentage of correct predictions (values range from 0% to 100%, with 1 representing optimum performance).

Sensitivity (recall): TP/(TP + FN) (values range from 0 to 1, with 1 representing optimum performance).

Specificity: TN/(TN + FP) (values range from 0 to 1, with 1 representing optimum performance).

Precision: TP/TP + FP (values range from 0 to 1, with 1 representing optimum performance).

F1 score: (2 × recall ×  precision)/(recall + precision) (values range from 0 to 1, with 1 representing optimum performance).

AUROC: area under the receiving operator characteristic curve (ROC), which plots the true positive rate (sensitivity) vs. the false positive rate (1—specificity) (values range from 0 to 1, with a value of 0.5 representing performance no better than random and a value of 1 representing optimum performance).

*TP = true positives, TN = true negatives, FP = false positives, FN = false negatives.

Accuracy as a metric has a high risk of bias given its dependence on the prevalence of a class in the population. For example, a model presented with 98 images of OL and 2 images of normal mucosa may accurately classify all the OL images and incorrectly classify all the normal mucosa images and still have 98% accuracy. The F1 score is known as the harmonic mean of sensitivity and precision and gives equal weight to FN and FP predictions (9). The F1 score can thus be advantageous in cases where classes are imbalanced, and as a single metric, it may be preferred over either sensitivity or specificity alone as prediction errors in one class might skew the sensitivity or specificity. The main drawback of the F1 score is that it does not consider TN predictions, but it is still applicable in scenarios where the goal is to minimize FN and FP (13). The AUROC can be useful in evaluating the discriminative performance of a classification model at various thresholds (9). AUROC values above 0.8 have typically been deemed to be clinically useful (14). It is important to note that while studies often showcase results with multiple performance metrics, the significance associated with a metric may depend on the image analysis question being studied, particularly as FN and FP predictions may not have the same implications across all clinical scenarios.

Object detection and segmentation tasks are often evaluated through a pre-defined threshold of intersection over union (IoU) between the pixels of the ground truth bounding box or mask and those of the model's prediction (9). A graph of sensitivity vs. specificity can be made, and the area under the curve will produce the average precision, another metric used in object detection and segmentation evaluation (15). The Dice Similarity Coefficient (DSC) has also been applied to semantic segmentation in particular (9, 15, 16). A spatial counterpart of the F1 score, the DSC is defined as 2(A∩B)/(A + B), or 2 times the intersection between the ground truth and the prediction divided by the total number of pixels of the two.

**Figure 1:**
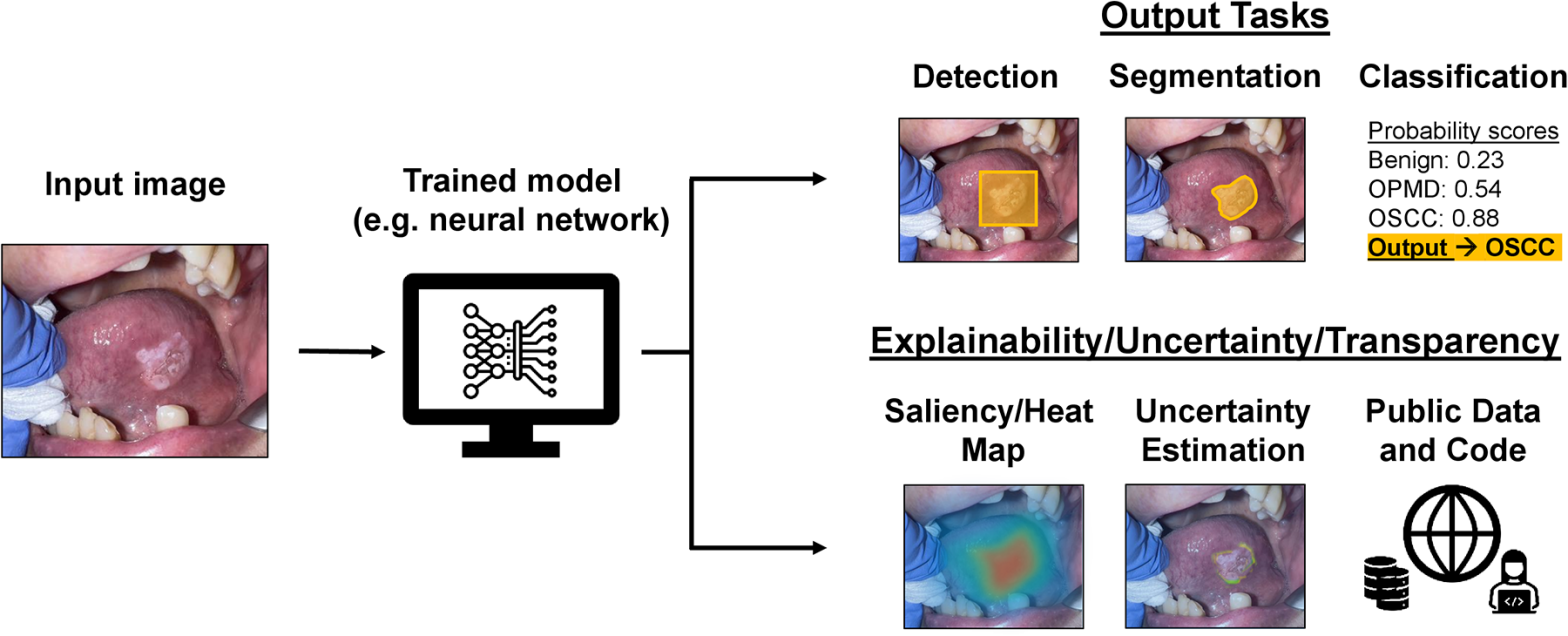
Possible applications and practices for AI models used for image analysis workflow of oral cavity clinical photographs. An oral cavity image can be fed as input into an already trained model, such as a neural network, and the resulting output (depending on for what and how the model was trained) could be for detection, segmentation, or classification of the lesion. There are also possible explainability, uncertainty quantification, and transparency practices that can be a part of this workflow to provide more context to the output of the trained AI model.

Regardless of performance outcomes, a persistent concern regarding the clinical application of AI models has been their lack of interpretability. Historically, AI models have been called “black boxes” due to the inability of humans to understand or reproduce how the models ultimately derive their predictions. *Explainability* refers to computational techniques aiming to elucidate the decision-making process of AI models, such as generating saliency maps that highlight the most important parts of an image used for its predicted classification (17). *Uncertainty quantification*, which refers to techniques aiming to estimate a model's level of confidence in its predictions, is another tactic to quantify the reliability of AI models (18). For example, in medical image segmentation tasks, probabilistic methods can generate uncertainty maps highlighting areas where the model is less confident in its delineation of anatomical structures or lesions. Together, explainability and uncertainty quantification techniques can offer insights into a model's decision-making and reliability, potentially increasing trust and acceptance of AI predictions in the clinical setting. Transparency in research, which can involve publicization of source code or data, can also facilitate progress in AI image analysis. Figure 1 demonstrates a possible workflow for AI image analysis of oral cavity photographs that incorporates explainability techniques, uncertainty quantification, and transparency practices.

## 3 Section 2: review of the literature on AI clinical photograph-based diagnosis of OSCC/OPMDs

A rigorous search of the literature was conducted through PubMed for studies on AI and the diagnosis of OSCC and/or OPMDs from clinical photographs published within the last 10 years. The keywords used, with appropriate Boolean operators, were: “artificial intelligence,” “deep learning,” “machine learning,” “diagnosis,” “detection,” “segmentation,” “classification,” “oral cancer,” “oral squamous cell carcinoma,” “oral potentially malignant disorders,” and “oral premalignant lesions.” Inclusion was restricted to peer-reviewed primary research studies on AI-based diagnosis of OSCC and/or OPMDs from clinical photographs, with exclusion of non-primary research studies, studies on imaging modalities other than clinical photographs, and studies in which AI diagnosis was based on analysis of clinical photographs and another imaging modality conjointly. Titles and abstracts of resulting papers were reviewed for relevance, followed by full-text review for inclusion and exclusion criteria. This process was repeated for relevant papers mentioned or cited within the studies meeting all inclusion and exclusion criteria. A total of 37 primary studies were ultimately identified and included in the review. Pertinent data from the studies included in the review were extracted into a spreadsheet, and the tabulated data is available in the Supplementary Table S1. For a structured summary and analysis of specific study characteristics, the review of the literature will be divided into the following sections: data sourcing; data processing and augmentation; ground truth establishment; image-analysis models and tasks; model training, testing, and validation; performance; explainability, uncertainty, and transparency.

### 3.1 Data sourcing

Computer vision studies for the detection of OSCC/OPMDs have used datasets of varying sizes, with the largest being the 6,903 photographs collected retrospectively by Hsu et al. (19). Many studies have specified the modality used for image capture, with smartphones and cameras being equally common. Figueroa et al. and Song et al. employed a custom mobile intraoral screening device for image collection that is also capable of integrating autofluorescence images (20–23). Some studies have obtained photos either wholly or in part from the Internet or from textbooks or scientific papers with less precise sourcing (15, 24–27).

Several authors have proposed standardized image-taking protocols to ensure consistency between datasets. Lin et al. (28) used smartphones and a grid-based center-positioning protocol for consistent lesion positioning and focal distance, suggesting that this method would allow automatic focusing by their AI model on discriminative disease regions without the need for the extra step of manual object localization (i.e., bounding box annotations by specialists). Kouketsu et al. (29) conducted one of the more standardized photography protocols, collecting photos taken by intraoral photography-trained oral surgeons via two Canon cameras, with constant 1:3 magnification, manual focus, maintenance of focus at a consistent distance from the subject, and maintenance of the lens perpendicular to the oral lesion of interest.

Given small sample sizes, the breadth and scope of OSCC/OPMD cases in study datasets have varied significantly. To better display the representativeness of their data, multiple authors have provided a more detailed breakdown of OSCC staging/grading, OPMD grouping (or presence/grade of OED), lesion anatomic locations, and/or other demographic and clinical metadata. Flügge et al. (30) included the breakdown of OSCC cases by stage, grade, and location. Fu et al. (31) included the breakdown of OSCC cases by stage and location and the breakdown of non-OSCC diseases by precise diagnosis. In both Flügge et al. and Fu et al., the T1 and T2 OSCC cases outnumbered the T3 and T4 OSCC cases. Kouketsu et al. (29) included the breakdown of tongue OSCC by stage (only including T1 and T2, as T3 and T4 were excluded) and the breakdown of benign tongue lesions by precise diagnosis. Tanriver et al. (15) included the breakdown of benign lesions by disease category. The full spectrum of OPMDs has not been investigated, with some disorders (i.e., oral lichenoid lesions, chronic graft-vs.-host disease, and others) not being included in any of the study datasets reviewed in this paper. Benign “lookalike lesions” (i.e., those with similar visual characteristics as OSCC/OPMD, such as traumatic ulcers and deep fungal infections) have rarely been included in datasets.

### 3.2 Data processing and augmentation

Following collection of study images, certain pre-algorithmic processing steps are performed, including image quality control, image normalization, and dataset harmonization (Table 1). Hsu et al. (19) delineated a scrupulous image quality control process, specifying the exclusion of intraoperative photos, postoperative photos, and photos including instrument-obscured parts or affected by stains or blurriness. Their images were deidentified by assignment of unique 6-digit padded number IDs. Normalization techniques such as resizing, cropping, color normalization, contrast enhancement, and noise reduction have been frequently applied. Alanazi et al. (32) used Gabor filtering to reduce noise, while Huang et al. (33) used a Noise Fading median filter to reduce noise.

In addition to their relatively small sizes, the datasets used for OSCC and/or OPMD image analysis have also in many cases suffered from imbalanced classes, reflecting the naturally differing prevalences and hence the photographic repositories of OSCC, OPMDs, and benign oral mucosal diseases in the populations studied. Such class bias has been acknowledged as a source for the propagation of errors in classification tasks (22). Various approaches have been employed at both the data level and algorithm level to address the overall lean datasets and the often-imbalanced classes noted therein. Data augmentation to increase dataset size has been applied in most studies. Notably, some researchers have included noise injection into photos as an augmentation technique to increase data variability and reproduce real-world imaging circumstances. This is in contrast to the pre-processing noise reduction techniques pursued by others (32, 33). While some studies have included augmented images in their test sets, others have refrained from testing augmented images, acknowledging that it is not possible to know if artefactual perturbation-specific characteristics could be embedded in images and subsequently reflected by the AI models in their decision-making outcomes. Several studies have not explicitly reported the use of any data augmentation techniques (16, 24, 29, 30, 32, 34–38).

To address imbalanced data, certain studies have applied random oversampling of minority classes with or without random undersampling of majority classes. With the goal of obtaining relatively even numbers of images in each class, random oversampling involves random selection of images to duplicate (from minority classes) while random undersampling refers to random selection of images to remove (from majority classes). Zhang et al., whose dataset contained 87 cancer images and 43 non-cancer images, used random oversampling to duplicate 44 non-cancer images to obtain 87 images in both classes (39). Lee et al. (40) introduced a novel data-level mosaic augmentation method that involved the creation of new distinct images containing a mosaic of representative areas from four other images, with greater areas allotted to underrepresented classes. Lee et al. noted improved performance with mosaic augmentation compared to random oversampling. At the algorithm level, Jubair et al. (34) applied weighted cross entropy loss, which grants more weight to minority classes when computing errors during training. Song et al. (22) variously tested weighted cross entropy loss, focal loss to reduce the influence of easily classified majority class images, and an ensemble (i.e., combination) of these algorithm-level approaches with random over- and undersampling techniques. Song et al. noted that the ensemble of cross entropy loss (algorithm-level) and over- and undersampling (data-level) led to improved overall performance compared to either algorithm-level or data-level techniques alone.

### 3.3 Ground truth establishment

Supervised learning has thus far been the standard for clinical image analysis in OSCC/OPMDs, rendering the establishment of ground truth annotations a critical step in study design. Ground truth annotations encompass all annotations made by human experts, which are used to train models in supervised learning and to gauge model performance. In classification tasks, ground truth annotations refer to the class label assigned to an image. Among studies on OSCC/OPMDs, this class label is typically the “diagnosis” for the lesion(s) in an image. In object detection and segmentation tasks, ground truth annotations comprise the manual localization of the lesion(s) in addition to the associated diagnoses.

Studies on OSCC/OPMDs have variously used histopathological and/or clinical ground truth diagnoses. For histopathological ground truth diagnoses, lesions would have been biopsied, and a definitive histopathological diagnosis rendered. For clinical ground truth diagnoses, lesions would have been given a diagnosis by clinician experts based on visual examination of the original lesion or an associated photograph. As previously mentioned, histopathology is the gold standard for diagnosis of OSCC and for diagnosis of dysplasia in OPMDs. However, certain “lower-risk” OPMDs or benign lesions may not undergo biopsy and histopathological examination, limiting the ground truth to their clinical diagnoses.

Histopathology has been almost universally used for the ground truth diagnosis of OSCC across image analysis studies. A number of studies, however, used a small (*n* = 131) dataset of OSCC and normal mucosal images publicly available on Kaggle, an online data science platform, and this dataset is not accompanied by any specification of histopathology for OSCC cases (32, 33, 36, 39, 41, 42). As expected, the diagnosis of OPMDs has been heterogeneous, with most studies using clinical diagnoses and only select studies using histopathological diagnoses for all or a subset of OPMDs. Many studies have employed experienced specialists for clinical diagnosis establishment, but few studies have explicitly mentioned the clinical diagnostic criteria adopted. Lee et al. (40) and Talwar et al. (37) specified diagnosis of OPMD based on the 2021 WHO consensus report on OPMD nomenclature and classification (2). Lin et al. (28) noted that histopathological diagnoses were available for certain “high-risk OPMDs” (eg., suspicious for dysplasia or OSCC) that happened to undergo biopsy during the course of their evaluation. Camalan et al. (43) and Kouketsu et al. (29) had histopathological ground truth diagnoses for all OPMDs as they restricted their analysis of OPMDs to dysplastic lesions (i.e., OL with various grades of OED). Ünsal et al. (16) and Warin et al. (44), on the other hand, had histopathological diagnoses for all OPMDs, which were not restricted to dysplastic lesions. Dinesh et al. (45) specified that histopathological diagnoses were available for all OSCC, OPMD, and normal mucosa images in their study.

Ground truth localization annotations comprise different types, and different types of localization annotations suit different tasks. Bounding box and polygonal annotations are typically used for object detection, while semantic and instance segmentation rely on more precise pixel-level masks. Bounding box annotations have sometimes been used in classification studies to supplement image-level diagnosis labels, with the suggestion that bounding boxes can provide an additional level of attention to a classification model by allowing it to focus on discriminative areas (20). Figueroa et al. (20), Hsu et al. (19), Parola et al. (46), and Welikala et al. (27, 47) used bounding box annotations in addition to diagnosis labels in their classification studies. Most object detection studies have used bounding boxes, but Keser et al. (35), Kouketsou et al. (29), and Dinesh et al. (45) used more precise polygonal annotations in their object detection studies. Song et al. (23), Ünsal et al. (16), and Vinayahalingam et al. (48) used pixel-level masks in their segmentation tasks. Few studies have explicitly proposed methods to address subjectivity in localization annotations. Vinayahalingam et al. (48) specified a calibration process and standardized annotation protocol for three clinicians performing pixel-wise annotations. Ünsal et al. (16), who had pixel-wise annotations performed by two specialists, calculated the intra-class correlation coefficient (ICC) for both inter- and intra-observer pixel-wise annotations and found them to be excellent. Warin et al. (44, 49) had bounding boxes for each target area drawn by three oral and maxillofacial surgeons, and the largest area of intersection between the separate boxes was taken as the ground truth. Welikala et al. (27, 47) proposed and employed a unique method of combining multiple clinicians’ bounding boxes into a single composite bounding box per lesion by first focusing on grouping and combining similar bounding boxes based on an IoU threshold and subsequently bringing bounding boxes together based on a criterion of simple overlap.

### 3.4 Image-analysis models and tasks

Several AI branches, most notably deep learning, have featured among studies aiming to diagnose OSCC/OPMDs from clinical photographs. Studies have applied specific computer vision tasks relevant to their research purposes, encompassing object detection (15, 19, 29, 33, 35, 40, 44, 46, 47, 49, 50), semantic segmentation (15, 16, 23), instance segmentation (15, 48), and classification. While the majority of studies focusing on classification have employed binary classification (20, 21, 25–27, 30–34, 36, 37, 39, 41–44, 47, 49, 51), multi-class classification has been assessed as well, up to 5 classes (15, 19, 22, 24, 26–28, 38, 40, 46, 48, 50, 52, 53). Certain studies have distinguished between OSCC and OPMDs, while others have merged OPMDs and OSCC into a single class given their overarching aim of separating lesions requiring referral or management from those not requiring the same. Hsu et al. (19), Lin et al. (28), and Welikala et al. (27) have all distinguished between higher-risk and lower-risk OPMDs in their multi-class classification experiments. However, there has been no standardization of risk stratification pursued among these studies, despite risk stratification for OPMDs being based on epidemiological data on risks for malignant transformation. For example, Hsu et al. (19) included oral submucous fibrosis in their middle or “yellow” referral urgency class together with oral lichen planus, and separate from either normal mucosa (low or “green” referral urgency) or non-homogeneous OL (high or “red” referral urgency), while Welikala et al. (27) categorized oral submucous fibrosis together with cancer in their highest referral class, the “cancer/high-risk OPMD” class. Vinayahalingam et al. (48) and Xie et al. (53) discriminated between two types of OPMD in their multi-class classification studies, specifically OL and oral lichen planus. However, no study to date has provided any more detailed discrimination among the eleven distinct groups of OPMDs beyond a dichotomous level.

Deep CNNs have featured as the most prevalent model in classification studies. Marzouk et al. (36) used a deep CNN for feature extraction followed by an auto-encoder for classification. An ensemble of deep CNNs has been used with improved performance in some classification studies (25, 46, 51). In addition to deep CNNs, swin transformers (21, 30, 37, 42, 48) and vision transformers (21, 37) have been applied to classification. Faster R-CNN (27, 44, 49), single shot multibox detector (29), and You Only Look Once (YOLO) (15, 19, 29, 49) have been used in object detection studies, while U-Net (15, 16) and Faster R-CNN (27, 44, 49) have been used in segmentation studies.

### 3.5 Model training, testing, and validation

Training a new model is an iterative process that requires a large amount of data and computational power. To overcome the issue of small datasets, transfer learning has been extensively used for model development, with adoption of AI models pre-trained on large datasets of images unrelated to the oral diseases of interest, in order to streamline the optimization of model parameters. Rabinovici-Cohen et al. (25) experimented with pre-training on medical data, specifically a skin lesion dataset, rather than standard non-medical transfer learning datasets, but found no improvement in their model's classification tasks with this approach.

Validation and testing have been approached differently in the literature. As previously described, validation is used during training to fine-tune the parameters of a model based on its performance, while testing (also termed internal or external validation depending on the test data's source) is the verification of a model's performance after all training and fine-tuning are finalized. A decrease in performance on the test data is expected, but the goal of testing is to gauge a model's ability to generalize to unseen data.

K-fold cross-validation is a type of internal validation technique used in some studies, in which the original dataset is split into *k* (number) equal, non-overlapping subsets. The model is then trained and tested *k* times, with each subset serving as the test set once while the remaining subsets form the training set. Importantly, the model is retrained from scratch in each iteration, using a different combination of subsets for training.

Other studies split their data into separate training/validation sets and a “held-out” test set (i.e., a set that is not used during the training process and only used to verify performance at the end). This approach also qualifies as internal validation.

Various studies have acknowledged that the relatively small photographic datasets used, often from a single institution or clinical department, may not reflect the full diversity of real-world clinical data. Multi-institutional images have been used in some studies (20–23, 27, 31, 43, 47). However, only 2 studies have actually pursued external validation to verify generalizability. Fu et al. (31), whose original dataset comprised photographs from 11 hospitals, tested their deep CNN classification model on a “clinical validation” set comprising 1,941 photographs from one hospital and an “external validation” set comprising 420 photographs from six dental and oral and maxillofacial surgery journals. Talwar et al. (37), who performed training and initial validation on a dataset drawn from community-based outreach programs of one institution, performed external validation using a dataset from a separate institution.

### 3.6 Performance

Studies published on OPMD and OSCC detection, segmentation, and classification have demonstrated overall favorable performance. Studies have variously described parameter optimization processes for enhancement of performance and in some cases tested various models against one another. A deeper analysis of the performance outcomes of these studies is beyond the scope of this paper, and interested readers are encouraged to refer to meta-analyses published on diagnostic performance of AI image analysis in OSCC/OPMD (54–56).

Various performance metrics have been reported in the literature. Among classification studies, the most commonly reported metrics have been accuracy, sensitivity (recall), specificity, precision, F1 score, and AUROC. In this context, accuracy refers to the percentage of correctly predicted class labels/diagnoses. Sensitivity (recall) represents the percentage of positive cases (ex: OSCC) correctly classified as positive. Specificity represents the percentage of negative cases (i.e., normal mucosa) correctly classified as negative. Precision represents the percentage of a model's positive classification outputs that are actually positive. The F1 score represents the harmonic mean of sensitivity and precision, granting equal weight to false positive and false negative outputs. In multi-class classification studies, these metrics have been calculated for each class.

Among classification studies, some of the highest performance outcomes are attested by Warin et al. (44), who achieved a sensitivity of 98.75%, specificity of 100%, F1 score of 0.99, and AUROC of 0.99 with their deep CNN classifier of OSCC vs. normal mucosa. The performance outcomes of the two studies that performed both internal and external validation will be highlighted. Fu et al., who developed a deep CNN for binary classification of OSCC vs. normal mucosa, achieved a sensitivity of 94.9%, specificity of 88.7%, and AUC of 0.983 on their internal test set and a sensitivity of 89.6%, specificity of 80.6%, and AUC of 0.935 on their external validation dataset (31). Talwar et al., who developed a deep CNN for binary classification of suspicious lesions (OPMD/oral cancer) vs. nonsuspicious lesions (benign lesions/normal mucosa), achieved a sensitivity of 0.83, specificity of 0.85, and F1-score of 0.86 on their internal test set and a sensitivity of 0.75, specificity of 0.70, and F1-score of 0.73 on their external validation dataset (37).

While AI could theoretically surpass humans in discriminatory capacity, few studies have compared AI and human performance in OSCC/OPMD diagnosis. Several studies have compared AI diagnostic performance to clinician experts and have failed to demonstrate the superiority of AI (25, 45). Fu et al. (31) did demonstrate superior performance of their classification model compared to non-experts (medical and non-medical students), but not experts (oral cancer specialists). Ye et al. (50) compared the performance of their classification model with that of senior-level clinicians, intermediate-level clinicians, and medical graduates specializing in oral and maxillofacial surgery, and they found that their model outperformed all three clinician groups in recall, precision, and specificity. Ye et al. also performed a multicenter field test of their model with separate external clinicians—specialists from dental hospitals, general dentists from general hospitals, and general dentists from community hospitals—and compared the change in performance of these clinicians when they classified images themselves and when they were subsequently provided the model's predictions as adjunctive diagnostic information. The authors found not only that the model significantly enhanced performance across all three groups, but also that it raised their performance in certain metrics beyond that of the senior-level dental clinicians tested before.

### 3.7 Explainability, uncertainty, and transparency

Of the studies reviewed here, only a third have incorporated explainability and interpretability tools for their AI models. In general, visual explanation methods such as saliency maps are very popular and of the studies reviewed here, the most common explainability tool has been a type of saliency map called the gradient-weighted class activation map, which transforms the photograph of interest into a gradient-based heat map that reflects the varying contribution of each pixel to the model's classification decision (25, 28, 30, 37, 40, 42–44, 47, 53). These maps have helped highlight certain pitfalls in AI models. For example, Figueroa et al. (20) noted that stained teeth in images were identified as discriminatory areas for OSCC/OPMD diagnosis, due to the coincidental presence of stained teeth in the OSCC/OPMD images (in the context of reported tobacco chewing) rather than true discernment of OSCC/OPMD visual characteristics.

Expanding upon the class activation map, Figueroa et al. (20) applied a guided attention inference network (GAIN) architecture for enhancing both their model's classification performance and its explainability. In this approach, they used traditional gradient-weighted class activation maps generated by their CNN after a classification task and reintroduced these maps as a separate input stream into the CNN, thereby guiding the network to focus its attention to discriminative areas of photographs.

Parola et al. (46) introduced a unique informed deep learning and case-based reasoning explainability system. The case-based reasoning approach uses similarity rankings between images to explain its decisions, which is said to reflect the decision-making process of clinicians as they diagnose new cases based on previously encountered similar cases. The model developed by Parola et al. provides a visual explanation for its outputs by presenting previous cases ranked by similarity to the input image. Parola et al. also introduced “informed learning” by having specialists produce their own similarity rankings to override the model's rankings in some cases, thereby training the model to better reflect human cognition.

Uncertainty quantification techniques aim to estimate the confidence of an AI model in its decisions. Uncertainty in AI models can arise from various sources, including data randomness or noise, model parameter uncertainty or architecture limitations, or inherent ambiguity in the task. Importantly, many classification models produce a probability score or inference score associated with the outputted class, but this is not necessarily associated with the uncertainty or confidence level of the model, as it may simply reflect the relative area of an image occupied by the pixels relevant to the classification. For example, Lee et al. (40) employed a soft labeling algorithm that involved the output of a result vector with three probability values representing the areas occupied by each of the three classes (OSCC, OPMD, and noncancerous lesions) in their classification study. Among the studies reviewed here, true uncertainty estimation has only been pursued by Song et al. (23), who developed a Bayesian deep network based on probabilistic rather than deterministic models for semantic segmentation of oral lesion images. This approach allows for the model to provide not only a predicted segmentation but also a measure of the model's confidence in that prediction, possibly enhancing clinical utility in OSCC/OPMD patient care.

Open science practices are crucial for increased transparency, reproducibility, and collaboration between researchers conducting medical AI model development. This includes making datasets, source code, and trained models publicly available to all. However, the clinical photograph datasets used in studies on OSCC/OPMD have rarely been made publicly available due to stated legal or ethical issues. Several groups have based their research on a publicly available dataset on Kaggle (131 images), but as previously mentioned, this dataset suffers from a small size, and a lack of specified histopathology and other clinical data that limit its validity (32, 33, 36, 39, 41, 42). Recently, Parola et al. (46) published their oral image dataset (567 images) with the stated goal of promoting research collaboration. Beyond datasets themselves, only a few select studies have published their source code for reference and use (28, 37, 46).

## 4 Section 3: discussion

Several limitations in study design and methodology become apparent upon a review of published studies on AI-based photograph analysis in OSCC/OPMD diagnosis. The most obvious and well-acknowledged is the small size of the clinical photograph datasets, even in comparison to other medical conditions or other imaging modalities. The limited breadth and scope of OSCC and OPMD photograph datasets present a hurdle for generalizability and clinical applicability, as it is prudent to conclude that few if any datasets currently available embody the full spectrum of OSCC, OPMDs, and benign lookalike lesions that may be encountered in a real-world clinical setting. Data augmentation, while necessary to create diversity in training data and prevent model overfitting, does not substitute for an independently larger dataset (10). Indeed, deep CNNs may be able to learn the original features of images despite augmentation techniques, diluting the novelty imparted by data augmentation. There is an urgent need for establishing a well-curated, reliable and representative database of clinical photographs of OSCC and OPMDs, ideally accompanied by clinical information and other metadata (57). Such a project has been initiated by Rajendran et al. (58) via the MeMoSA® platform and Piyarathne et al. (59), but only the latter's repository is currently publicly available.

A large-scale, public database on the order of tens to hundreds of thousands of trustworthy images could not only be used as external validation for future studies, but also possibly even facilitate the self-supervised training of foundation models that can learn generalizable representations of OSCC/OPMD clinical images. While amassing a repository of that scale of is a non-trivial pursuit, even having a central database could facilitate the fine-tuning of other already published natural image foundation models or medical image foundation models for OSCC/OPMD clinical image purposes. Fine-tuning trained foundation models on smaller datasets for specific tasks like OSCC/OPMD detection and segmentation, can potentially improve model performance, robustness, and generalization while reducing the amount of task-specific training data required. This approach has become quite popular in the analysis of many other medical images, from histopathology whole-slide images to radiology 3D image stacks and even dermatological clinical images.

As far as supervised learning is concerned, external ground truth establishment is critical, which introduces another potential pitfall in studies given the subjectivity of ground truths. Indeed, clinicians’ diagnoses of OPMDs based on visual examination may suffer from intra-observer and/or inter-observer heterogeneity (60). Discordance in labeling and annotation of clinical images can transfer human subjectivity to AI models, which can propagate this issue. As previously discussed, multi-sourced annotation of individual images has been proposed to reduce bias and has been employed in some studies with varying methods of resolution of inter-observer disagreement (16, 27, 44, 47, 49). There is no consensus on optimal techniques for resolution of disagreement or integration of multiple localization annotations, as the attested techniques carry different risks of FN and FP outcomes that must be weighed with respect to the clinical implications of these in the context of the clinical questions studied.

The impact of image-taking modality, image quality, and image normalization have yet to be studied in OSCC/OPMD photograph analysis. It is naturally ideal to develop a model that can process photographs derived from varying modalities and that is resistant to image artefacts and variances. A model trained on a large, robust dataset may be invariant to minor discrepancies in quality and normalization. Given small datasets, however, diligent quality control and normalization may be required in the data preparation phase to minimize the propagation of biases and errors. Nonetheless, from a clinical applicability perspective, models trained on perfected images may not be generalizable to real-world photographs. Image dataset harmonization is distinct from image quality improvement. Certain steps to clean and organize the dataset such as case anonymization, removal of sensitive information, extraction of useful labeling information, unique labeling, and standardized annotation should be pursued to safeguard data privacy and minimize logistical errors (61). Scrupulous data maintenance and harmonization can increase the feasibility of amalgamating image datasets for training or testing and facilitate multi-institutional collaboration (62, 63).

Careful evaluation of a study's aim, computer vision task studied, and study design is also important in assessing clinical applicability. Given the great variance in objectives pursued among existing studies, not every model developed will necessarily be meaningful in clinical OSCC and OPMD diagnosis. For example, some studies have sought to classify OSCC vs. normal mucosa, which is not particularly meaningful for clinical practice unless early-stage cancers without overt visual features of OSCC can be detected with high accuracy akin to advanced cancers. Indeed, fewer studies have focused on the “grey” areas, such as differentiation of OSCC or OPMDs vs. benign look-alikes, early stage OSCC vs. OPMDs, or high-risk OPMDs vs. low-risk OPMDs. In a primary care setting, introducing an image of a benign, chronic traumatic ulcer with a “suspicious” appearance into a binary classifier of OSCC vs. normal mucosa may lead to a grossly inaccurate result. Binary classification systems distinguishing OSCC and high-grade dysplasia vs. low-grade dysplasia and benign look-alike lesions would be more useful than binary classification of OSCC vs. normal mucosa. In the specialist setting, the development of a multi-classification system for the diagnosis of the full taxonomy of oral mucosal pathologies may prove to be more clinically meaningful. It will also be important to further investigate the performance and scope of AI image analysis in OPMDs that rely more prominently on tactile examination for accurate diagnosis, namely oral submucous fibrosis. The ideal AI model will reflect the ontology used in clinical practice for OSCC/OPMD diagnosis and management, and its model architecture should be carefully designed considering the dataset characteristics, sample size, and desired capabilities. This includes incorporating explainability and/or uncertainty quantification to enhance clinical usefulness. In any case, selection of a clinically meaningful question should be at the forefront of AI image analysis study design, and oral medicine specialists can help drive future research in valuable directions in conjunction with other clinicians and data scientist experts. Additionally, conducting real-world implementation studies to evaluate the impact of AI systems on clinical outcomes, workflow efficiency, and cost-effectiveness in various healthcare settings would provide valuable insights into the practical benefits and potential limitations of AI-assisted diagnosis.

Many studies have shown promising performance statistics, and the majority of studies have aptly presented well-established core metrics such as accuracy, sensitivity, specificity, F1 score, and AUROC. No single metric embodies all essential properties of a model's performance, hence the broad array of metrics reported in the literature (64). Moreover, it is important to note that the relative significance attributed to a metric may differ based on the setting in which a model is applied. For example, FP outcomes for OSCC or OPMD diagnosis may have differing implications in a primary care setting compared to a specialist setting, and in a low-resource setting compared to a high-resource setting. Despite these nuances, sensitivity has been noted by some to be the critical metric in oral cancer diagnostics given the greater consequences associated with a high FN rate (65). Another important consideration is how to determine what values for performance metrics are clinically acceptable. There are no consensus guidelines on acceptable values for sensitivity and specificity in OSCC/OPMD diagnostics. However, the current gold standard, i.e., conventional oral examination performed prior to histopathological analysis, has been found to have sensitivity ranging from 0.5–0.99 and specificity ranging from 0.94–0.99 (66). It has been suggested that novel diagnostic systems must have sensitivity and specificity values above 0.9–0.95 to prove clinically superior to the conventional oral exam (4). Rigorous controlled trials comparing the performance of AI models and human experts, and as per Ye et al. (50), comparing the changes in human expert performance when unaided vs. when aided by AI models, can allow for better contextualization and validation of AI diagnostic performance.

Regardless of high performance, the risk of a model not being able to generalize to real-world data is ever present as long as representative datasets are not available for training and more extensive external validation is not performed. Few studies on OSCC OPMD image analysis have pursued external validation, which hampers their current clinical applicability. The comparison of the performance of various models seen in many studies also warrants additional scrutiny. While it is common in the field of data science to test the performance of various algorithms or models against one another, the comparison of models and the selection of one based on its performance on an internal test set essentially relegates that test set to a validation set (10). In this context, the differences in performance outcomes among models noted may be of questionable value. Future research should prioritize developing and validating models across multiple diverse datasets to establish both broad applicability across institutions and geographic regions, as well as to define the specific conditions under which these models perform reliably. This approach, combined with thorough external validation, will help bridge the gap between promising research results and meaningful clinical implementation.

The need for developing explainable AI has been stressed for some time, yet progress in this domain has been slow. The primary explainability technique attested in OPMD and oral cancer photograph analysis is the class activation map, which provides a visual survey of the relative discriminative utility of each image region or pixel to the model's prediction. Only one study reviewed here has incorporated an example-based explainability approach (46). In addition, the paucity of studies reviewed here that have presented uncertainty quantification remains another obstacle to AI incorporation into high-stakes medical decision-making. To advance the community of AI model development for clinical photographs of OSCC/OPMD, future studies should prioritize exploring both explainability and uncertainty quantification methods (e.g., SHapley Additive exPlanations, feature attribution methods, Bayesian neural networks, Monte Carlo dropout, etc.) that are commonly employed in other similar medical image analysis subfields (67, 68).

Headway in AI image analysis depends on greater transparency in research. Several studies on OSCC/OPMD have published their source codes, while few have published their image datasets. Deliberate efforts to navigate relevant ethical and legal requirements during initial study design can allow safe data publicization and sharing, which in turn will provide an impetus in the development of standardized and representative databases of OSCC/OPMD images for external validation. These efforts should begin with obtaining informed consent during data collection from all patients, with explicit mention that images will be used for medical image analysis and discussion of the possible risks and benefits. Furthermore, protocols for de-identification of images and maintenance of privacy should be established. Even publishing only the source code of the model architecture and training protocols should be standard practice for reproducibility and greater transparency. Recently, the TRIPOD + AI statement was published to provide recommendations on responsible reporting for studies describing the development and evaluation of a clinical AI prediction model (69). This will further encourage the practice of open science in the field of medical AI image analysis.

While computer vision in the study of OSCC/OPMD has thus far focused on images, video analysis may be a feasible future direction. CNNs have been used to study pharyngeal cancer diagnosis from endoscopic videos (70). The development of a standardized video-taking protocol could offer an opportunity for real-time screening of the oral cavity for OPMD and oral cancer. *Multi-modal AI*, which can integrate and process data from multiple modalities, such as images, text, and audio, has the potential to expand AI's diagnostic and predictive power beyond that of image-based analysis. Just as clinicians rely on more than a visual examination to render a diagnosis of OSCC/OPMD, multi-modal AI systems will likely prove more efficacious by synthesizing clinical imaging with other data sources such as patient history, demographic information, histopathology images, and/or videos. ChatGPT, a generative and multimodal AI system, has recently been studied for its ability to diagnose of oral mucosal pathology based on visual and textual data (71). Multi-modal approaches to OSCC/OPMD clinical image analysis can potentially improve diagnostic accuracy. Additionally, developing models that can analyze sequences of images over time to detect progression of OPMDs or early signs of malignant transformation could be particularly valuable for monitoring high-risk patients and identifying subtle changes that may indicate disease progression.

As AI continues to broaden in scope and potential in OSCC/OPMD diagnostics, clinicians such as oral medicine specialists can assist in study design to engender meaningful progress in the field. Such specialists who are involved in OSCC/OPMD management can contribute significantly to the standardization of clinical photograph capture and enrichment of potential future public repositories of OSCC/OPMD/benign lookalike images from diverse populations. Similarly, they can contribute to projects involving multi-sourced annotation of images and provide valuable insight to data scientists on designing AI models that can synthesize multiple data modalities and explain their prediction and confidence in that prediction. Importantly, specialists will be able to define the characteristic spectrum of disease (OSCC/OPMD/benign lookalike lesions) as well as the clinical dilemmas in management. This will help guide relevant and ethical study questions appropriate to the clinical setting (ex: primary vs. secondary, geographic location) and ensure concordance with the ontologies, nomenclature, and diagnostic criteria used in clinical practice. This will also bring into relief the desiderata for representative external validation sets, narrowing the gap toward clinical applicability. Table 2 summarizes the primary limitations of the current state of OSCC/OPMD image analysis and proposes action plans for improvement, many of which can be supported by global oral medicine.

**Table 2:** Primary limitations of current OSCC/OPMD AI image analysis studies and proposed action plans for improvement.

Current limitations in OSCC/OPMD AI image analysis	Action plans for future research
Limited size, diversity, and representativeness of image datasets	Global, multi-institutional collaboration to establish a large, variegated repository of OSCC/OPMD images as well as benign lookalike lesions, ideally accompanied by clinical metadata
Subjectivity and variability in ground truth establishment	Adoption of gold standard criteria for ground truth diagnoses (histopathology for OSCC, consensus diagnostic criteria for OPMDs) and protocols to resolve intra-observer and inter-observer heterogeneity for ground truth annotations
Questionable clinical meaningfulness of study aims	Selection of clinically meaningful study questions applicable to the clinical setting (ex: primary vs. secondary) and population of interest, through collaboration with clinicians and other stakeholders
Limited external validation	External validation with image sets that represent the population of interest
Limited explainability	Further research in data engineering to develop explainability methods that allow interpretation of AI model decisions by clinicians and other stakeholders
Limited uncertainty quantification	Further research in data engineering to develop methods of quantifying confidence or uncertainty of AI models in their decisions
Limited transparency in research	Working in accordance with ethico-legal frameworks to make feasible the public sharing of image repositories and source codes for research advancement

Ultimately, AI image analysis for OSCC/OPMD diagnosis is still in the nascent phase of technical feasibility, and further methodological advancements and rigorous study design enhancements are needed to be able to establish generalizability and clinical applicability. Beyond clinical applicability, implementation and workflow as well as actual patient benefit must be considered. This review infers that AI is currently incapable of replacing a conventional visual and tactile exam in the OSCC/OPMD diagnostic process. Well-designed prospective studies may help establish clinical generalizability and clinical applicability, and randomized controlled trials comparing AI either alone or in adjunctive capacity to conventional diagnostic methods will be necessary to gauge the benefit to risk ratio of AI image analysis. This requisite pathway underlines the crucial need for closer collaboration between clinicians, data scientists, and other stakeholders in future research on AI image analysis for OSCC/OPMD diagnosis.
